# Semaglutide in the treatment of a patient with type 2 diabetes mellitus, psoriasis, and metabolic dysfunction-associated steatotic liver disease: a case report

**DOI:** 10.3389/fmed.2025.1684204

**Published:** 2025-09-12

**Authors:** Bingting Lin, Qiuxiang Huang, Liang Weng, Lu Lin

**Affiliations:** Fuzong Clinical Medical College of Fujian Medical University, 900th Hospital of PLA Joint Logistic Support Force, Fuzhou, China

**Keywords:** type 2 diabetes mellitus, psoriasis, semaglutide, a case report, metabolic dysfunction-associated steatotic liver disease

## Abstract

This case report highlights the therapeutic efficacy of semaglutide in a 49-year-old male with concurrent type 2 diabetes mellitus (T2DM), psoriasis (PASI 22), and metabolic dysfunction-associated steatotic liver disease (MASLD). The patient presented with metabolic dysregulation, severe psoriatic lesions, and hepatic steatosis. Initial therapies, including acitretin and topical agents, failed to yield clinical improvement. Semaglutide was initiated at 0.25 mg/week, titrated to 1 mg/week over 12 weeks, alongside metformin dose adjustment. At 24 weeks, the patient exhibited significant improvements: HbA1c decreased from 9.8 to 7.2%, body weight reduced by 6 kg, liver enzymes returned to normal ranges, and PASI declined to 6.2. Treatment was well-tolerated with no severe adverse events.

## 1 Background

Type 2 diabetes mellitus (T2DM), psoriasis, and metabolic dysfunction-associated steatotic liver disease (MASLD) are common chronic conditions (1), and certain patients may present with a combination of these three diseases in clinical practice. Their coexistence suggests shared pathogenic mechanisms, including chronic inflammation, insulin resistance, and metabolic dysregulation (2). In recent years, semaglutide, a novel glucagon-like peptide-1 (GLP-1) receptor agonist administered as a once-weekly injection, has demonstrated significant efficacy in improving glycemic control, promoting weight loss, and alleviating hepatic steatosis (3). Previous research by our team found that liraglutide, a GLP-1 receptor agonist with lower receptor affinity, can improve psoriasis symptoms in diabetic patients by inhibiting inflammatory pathways (4). Given semaglutide’s stronger GLP-1 receptor binding, its immunomodulatory effects may be more pronounced. Emerging evidence also supports the role of semaglutide in other inflammatory skin diseases such as hidradenitis suppurativa (5). Although the efficacy of semaglutide in treating individual metabolic diseases has been reported, this case study demonstrates its combined benefits in patients with all three comorbidities. Patients with such conditions typically require polypharmacy, but the pleiotropic effects of semaglutide may simplify treatment regimens and reduce the risk of drug interactions. This finding highlights a novel approach to managing complex metabolic syndromes.

## 2 Diagnostic process

### 2.1 Patient characteristics and physical examination

A 49-year-old male presented on May 3, 2023, with no significant medical, personal, or familial history. Physical examination revealed the following:

*Anthropometrics*: Height 167 cm, weight 73 kg, body mass index (BMI) 26.18 kg/m^2^.
*Dermatologic findings*: Diffuse infiltrated plaques and papulonodular lesions on the trunk, limbs, and face; scattered nodules on extremities; adherent white scales with collar-like desquamation. Facial lesions measured approximately 5–10 mm in diameter (green bean-sized). Critically, the patient developed widespread sterile pustules on an erythematous base across the torso and limbs, alongside large yellowish plaques with scattered pustules on the soles.
*Nail changes*: Grayish-yellow discoloration, thickened nail plates, and marked periungual hyperkeratosis.
*Disease severity*: Psoriasis Area and Severity Index (PASI) score 22; Dermatology Life Quality Index (DLQI) 27. The constellation of plaque psoriasis with acute, widespread pustulation was highly suggestive of a concomitant generalized pustular psoriasis (GPP) flare, complicating his underlying plaque-type psoriasis.

### 2.2 Laboratory and auxiliary examinations

**Figure 1:**
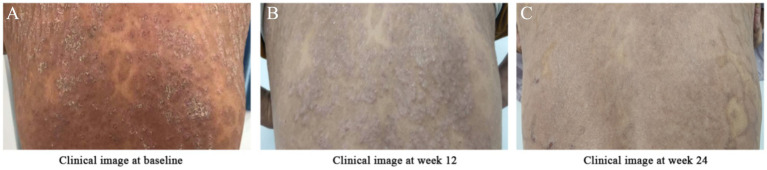
Changes in psoriatic lesions on the patient’s back at baseline (pre-semaglutide), Week 12, and Week 24 of semaglutide treatment **(A–C)**. **(A)** Baseline (pre-treatment): Diffuse infiltrated plaques and papules on the trunk with adherent white scales and collar-like desquamation. **(B)** Week 12 post-semaglutide: Gradual resolution of pustules, shedding and shrinkage of psoriatic plaques, and significant improvement in pruritus. **(C)** Week 24 post-semaglutide: Near-complete resolution of plaques and scales; reduced limb pain and improved mobility.

**Figure 2:**
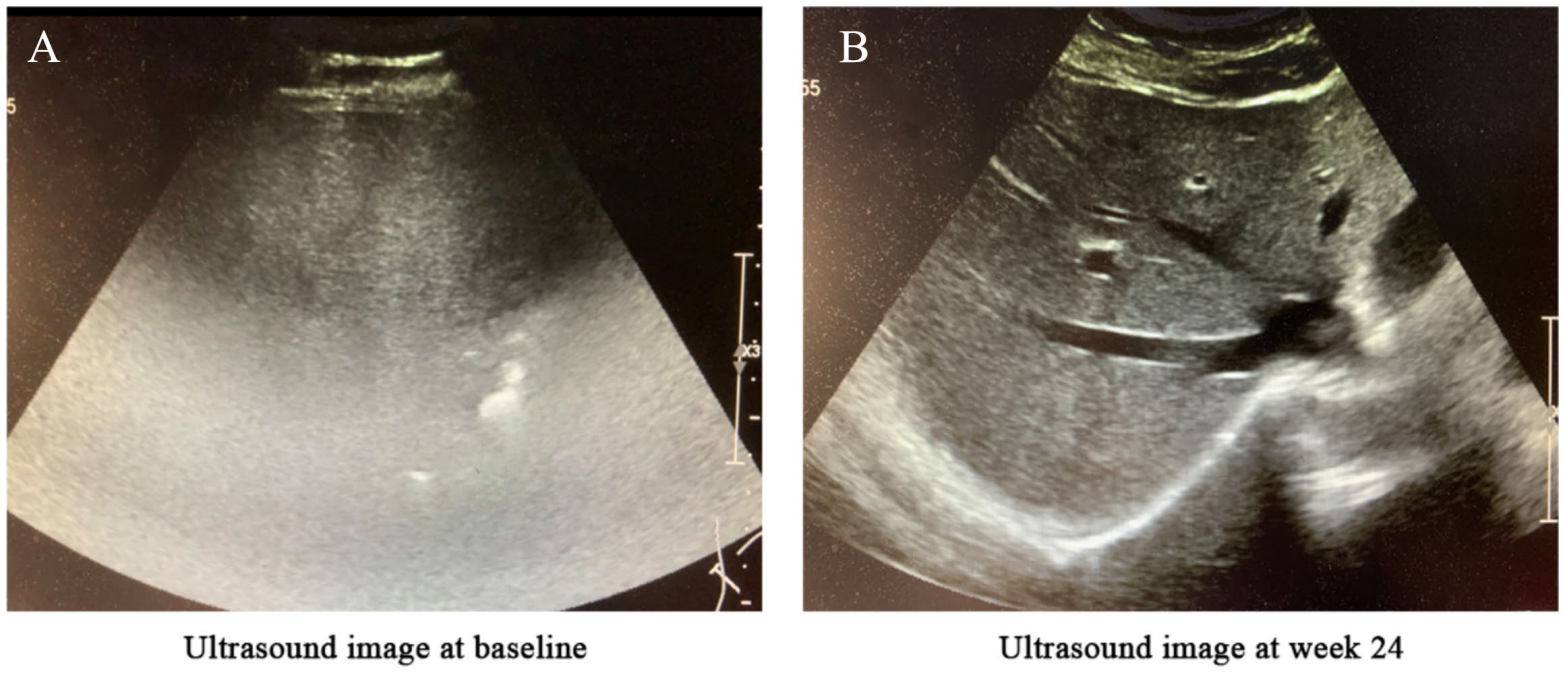
Changes in liver ultrasound imaging before and after treatment. **(A)** Baseline (before treatment): The hepatic parenchyma exhibited diffusely enhanced echogenicity, with unclear or absent visualization of intrahepatic blood vessels and significant attenuation of far-field echoes. The diaphragm was not visualized, suggesting severe fatty liver. **(B)** After 24 weeks of semaglutide treatment: The hepatic parenchyma showed diffusely increased echogenicity; however, the diaphragm and intrahepatic blood vessel walls were clearly visualized, indicating mild fatty liver.

*Glycemic Metabolism Indicators*: The patient’s glycated hemoglobin (HbA1c) was 7.8%. Fasting blood glucose (FBG) was 8.5 mmol/L, and 2-h postprandial blood glucose (2 h-PBG) was 11.3 mmol/L. Fasting insulin (INS) was 15.18 μU/mL, and fasting C-peptide was 3.16 ng/mL. Testing for diabetes-related autoantibodies—including insulin autoantibody (IAA), islet cell antibody (ICA), and glutamic acid decarboxylase antibody (GADA)—yielded negative results.
*Lipid Metabolism Indicators*: Total cholesterol (TC) was 5.28 mmol/L, triglycerides (TG) were 2.4 mmol/L, high-density lipoprotein cholesterol (HDL-C) was 0.63 mmol/L, and low-density lipoprotein cholesterol (LDL-C) was 3.82 mmol/L. Inflammatory Marker: C-reactive protein (CRP) was elevated at 17.3 mg/L. Liver Function Indicators: Alanine aminotransferase (ALT) was 58 U/L, and aspartate aminotransferase (AST) was 48 U/L. Other.
*Examinations*: Skin biopsy confirmed psoriasis (Figure 1A). Abdominal ultrasound revealed severe MASLD (6) (Figure 2A).

## 3 Treatment plan

Based on the patient’s clinical presentation and auxiliary examinations, a definitive diagnosis of T2DM, psoriasis, and MASLD was established. Initial treatment included acitretin (to regulate keratinization), topical tretinoin cream, sodium thiosulfate (for pruritus relief), and metformin (1,000 mg daily for glycemic control). However, psoriasis symptoms showed no significant improvement, and the disease progressed rapidly, with widespread pustules and desquamation involving the entire body (Figure 1A).

On May 17, 2023, semaglutide was initiated at a starting dose of 0.25 mg/week, combined with continued metformin (1,000 mg daily). This time point is designated as ‘Baseline’ for all subsequent efficacy and safety assessments. Acitretin and other medications were discontinued. The semaglutide dose was escalated to 0.5 mg/week after 4 weeks and further to 1 mg/week at 8 weeks. Mild transient nausea occurred during dose escalation (0.5 → 1 mg/week at Week 8–10), lasting ≤48 h without vomiting. No antiemetics were required. Topical tretinoin cream was maintained for psoriasis symptom management.

### 3.1 Treatment outcomes

**Figure 3:**
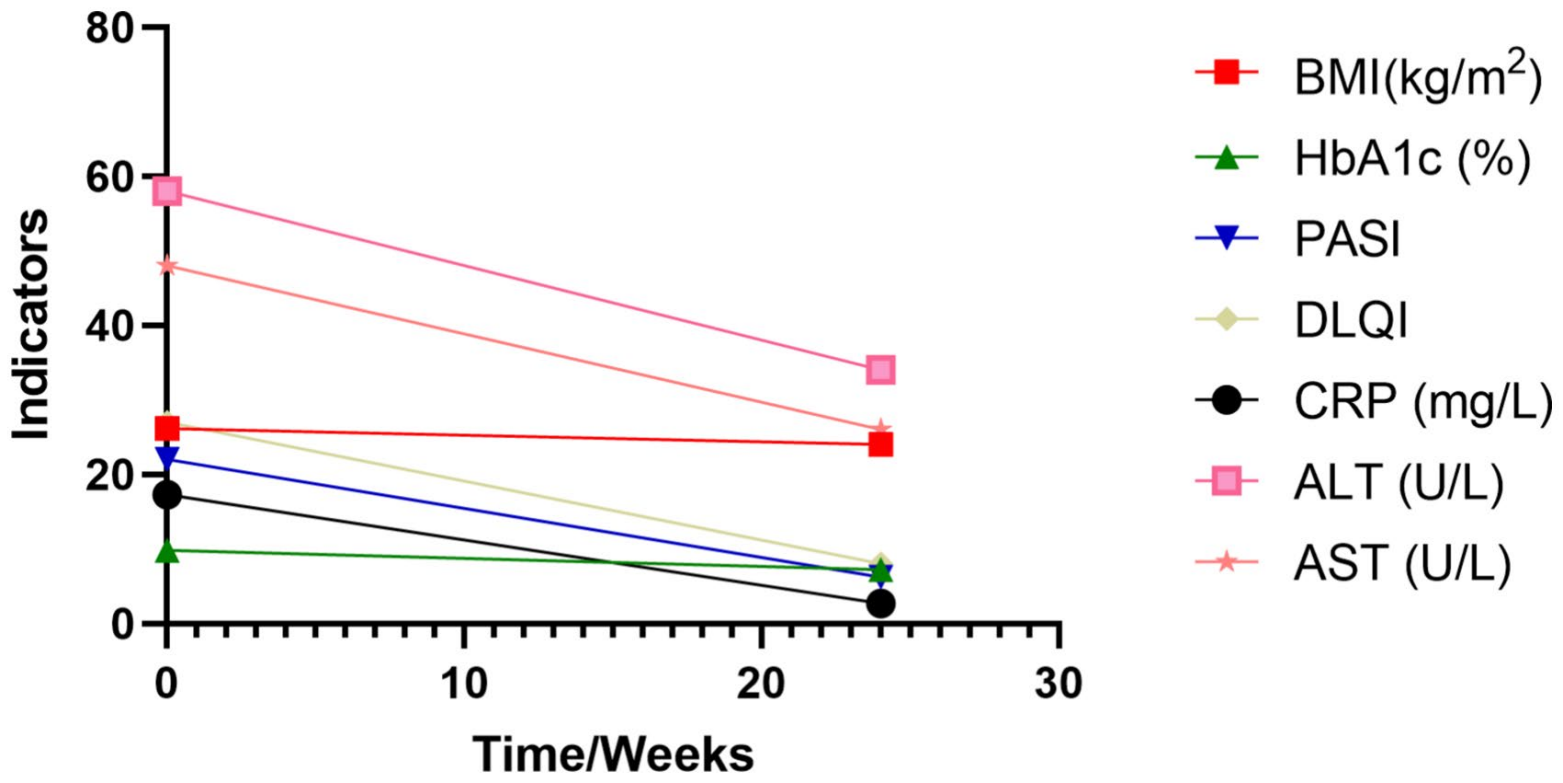
Changes in key indicators before and after treatment. BMI, Body Mass Index; HbA1c, glycated hemoglobin; PASI, Psoriasis Area and Severity Index; DLQI, Dermatology Life Quality Index; CRP, C-Reactive Protein; ALT, Alanine Aminotransferase; AST, Aspartate Aminotransferase.

*Week 12*: Weight loss of 4 kg, gradual resolution of pustules, shrinkage of psoriatic plaques, and marked improvement in pruritus (Figure 1B).
*Week 24*:
Metabolic parameters: HbA1c decreased to 6.5%, body weight reduced by 6 kg, BMI 24.02 kg/m^2^.
Liver function: ALT declined from 58 to 48 U/L; AST from 48 to 26 U/L (Figure 3).
Dermatological response: Near-complete resolution of psoriatic lesions and pruritus (Figure 1C).
Hepatic imaging: Abdominal ultrasound demonstrated reduced hepatic steatosis (Figure 2B).

The regimen was well-tolerated, with only mild gastrointestinal discomfort reported. No hypoglycemia or severe adverse events occurred. The patient reported high satisfaction with the treatment outcome (Table 1).

**Table 1:** Changes in various indicators from Baseline (pre-semaglutide) to Week 24 of semaglutide treatment.

Indicators	Baseline	Week 24	Reference range
Body Weight (kg)	73	67	N/A
Body Mass Index (BMI, kg/m^2^)	26.18	24.02	18.5–24.9
Waist Circumference (cm)	107	101	<90 (Asian Men)
Systolic Blood Pressure (mmHg)	127	123	<139
Diastolic Blood Pressure (mmHg)	80	82	<90
HbA1c (%)	9.8	7.2	4.0–6.0
Fasting Insulin (INS, μU/mL)	15.18	11.77	2.6–24.9
C-Peptide (ng/mL)	3.16	3.62	1.1–4.4
Fasting Blood Glucose (FBG, mmol/L)	8.5	7.8	3.9–6.1
2 h Postprandial Blood Glucose (2 h-PBG, mmol/L)	11.3	9.6	<7.8
HOMA-IR	5.74	4.08	<2.5
Psoriasis Area and Severity Index (PASI)	22	6.2	0
Dermatology Life Quality Index (DLQI)	27	8	0
C-Reactive Protein (CRP, mg/L)	17.3	2.7	0–3
Alanine Aminotransferase (ALT, U/L)	58	48	0–40
Aspartate Aminotransferase (AST, U/L)	34	26	0–40
Total Cholesterol (TC, mmol/L)	5.28	4.36	<5.2
Triglycerides (TG, mmol/L)	2.4	0.78	<1.7
HDL-Cholesterol (HDL-C, mmol/L)	0.63	0.82	>1.0 (Men)
LDL-Cholesterol (LDL-C, mmol/L)	3.82	3.01	<3.4

## 4 Discussion

This case report demonstrates the multifaceted therapeutic efficacy of semaglutide in a patient with T2DM, psoriasis, and MASLD. Prior to treatment, the patient exhibited significant metabolic dysfunction accompanied by severe dermatological manifestations (PASI: 22; DLQI: 27), indicative of severe psoriasis with concurrent insulin resistance and fatty liver disease. After 24 weeks of semaglutide treatment, the patient demonstrated notable improvements in body weight, waist circumference, glucose, and lipid metabolism parameters. This case highlights semaglutide’s potential as a therapeutic option for patients with complex metabolic-immune comorbidities. Its advantages of reducing polypharmacy and improving treatment adherence align with the principles of personalized precision medicine.

One of the most striking outcomes was the improvement in psoriasis symptoms, including the rapid resolution of widespread pustules indicative of a GPP flare. This case adds to the growing evidence of semaglutide’s benefit in patients with metabolic-inflammatory comorbidities. A critical consideration is whether the dermatological improvement stems primarily from a direct immunomodulatory effect of semaglutide or is secondary to the substantial weight loss and metabolic improvements achieved. Obesity is known to exacerbate psoriasis and impair treatment response, mediated by chronic inflammation from adipose tissue (7). The observed weight loss and metabolic enhancements undoubtedly created a less pro-inflammatory systemic environment, which likely contributed significantly to the skin clearance. However, the rapidity and degree of response, particularly following failure of prior conventional therapy, may also suggest additional, direct anti-inflammatory properties of semaglutide. The core pathogenic mechanism of psoriasis involves abnormal Th17 cell activation and dysregulation of the IL-23/IL-17 signaling pathway (8). Semaglutide may exert its effects through three key mechanisms: (1) direct inhibition of IL-23 secretion and Th17 cell differentiation; (2) enhancement of insulin sensitivity and reduction of proinflammatory cytokines; (3) attenuation of hepatic fat deposition and suppression of hepatic stellate cell activation. This multitarget action makes semaglutide particularly suitable for metabolic-immune comorbidities. In this case, the marked reduction in CRP (17.3 → 2.7 mg/L), improved insulin resistance (HOMA-IR: 5.74 → 4.08), and resolution of hepatic steatosis (ALT: 58 → 48 U/L; AST: 48 → 26 U/L) corroborate these mechanistic pathways.

Regarding MASLD, semaglutide’s benefits were equally notable. The disease’s pathogenesis centers on insulin resistance, hepatocellular lipid accumulation, and chronic inflammation. Semaglutide has been shown to ameliorate hepatic steatosis through weight loss, insulin sensitization, and direct reduction of hepatic fat content (9). In this patient, ultrasound findings confirmed transition from severe to mild MASLD, paralleling improvements in liver enzymes and metabolic parameters.

A key challenge in clinical practice is managing patients with multisystem metabolic disorders. Conventional approaches targeting individual diseases often fail to address their interconnected pathophysiology. This is particularly relevant for psoriasis, a chronic inflammatory condition with profound quality-of-life implications. While semaglutide’s efficacy in isolated conditions [e.g., T2DM, MASLD, or psoriasis (10)] is established, a recent clinical study demonstrates that its systemic anti-inflammatory properties confer benefits in patients with comorbid psoriasis and obesity-related type 2 diabetes (10). Our report to document its synergistic efficacy in simultaneously controlling these three metabolic and immune comorbidities. Semaglutide’s ability to simplify complex treatment regimens—reducing polypharmacy, minimizing adverse event risks, and enhancing adherence—represents a paradigm shift in managing such patients.

Overall, this case provides novel insights into semaglutide’s role in complex metabolic disorders. Crucially, this report should not be interpreted as evidence for semaglutide as a first-line monotherapy for psoriasis. Instead, it underscores the importance of addressing underlying metabolic dysfunction, including through weight management and lifestyle modifications, as a cornerstone of care for patients with psoriasis and metabolic comorbidities. Future large-scale, multicenter studies are needed to validate semaglutide’s long-term efficacy and safety in comorbid populations. Although the current observation period is limited to 24 weeks, the patient remains on semaglutide, with a planned 1-year follow-up to assess durability of response. Importantly, the risk of symptom rebound after discontinuation remains unexplored and warrants further investigation.

## Data Availability

The original contributions presented in the study are included in the article/supplementary material, further inquiries can be directed to the corresponding author.
